# Bridging brain, respiratory, and locomotor muscle: Integrated view of between‐sex differences during exercise

**DOI:** 10.1113/EP093488

**Published:** 2025-11-29

**Authors:** Tomasz Kowalski, Soichi Ando

**Affiliations:** ^1^ Department of Physiology Institute of Sport‐National Research Institute Warsaw Poland; ^2^ Graduate School of Informatics and Engineering The University of Electro‐Communications Tokyo Japan

**Keywords:** athletic performance, exercise physiology, near‐infrared spectroscopy, NIRS, oxygen saturation, oxygenation

1

Understanding sex‐based physiological differences in exercise performance remains an important challenge in sports science. While previous research has emphasized differences in maximal oxygen uptake, ventilatory function and peripheral fatigue, few studies have simultaneously examined cerebral and muscular oxygenation responses during exhaustive exercise. The study by Ramos‐López et al. (2025) provides a novel and valuable contribution by applying near‐infrared spectroscopy (NIRS) to comprehensively characterize oxygenation kinetics in the prefrontal cortex, respiratory and locomotor muscles during incremental cycling to exhaustion in endurance‐trained individuals (Ramos‐López et al., 2025). The authors observed that males showed greater deoxygenation in locomotor muscles and higher oxygenation in the prefrontal cortex, whereas females exhibited higher oxygenation in respiratory muscles and comparatively lower brain oxygenation responses. These findings reveal sex‐specific patterns of tissue oxygenation during exhaustive exercise, which the authors interpret as possible evidence of differing limitations to performance. Consequently, females may experience greater central limitations, reflected in altered oxygenation of cognitive–motor control regions, whereas males appear more affected by peripheral factors.

Grounding their interpretation in established physiological mechanisms, the authors draw on evidence related to the respiratory metaboreflex and cerebral oxygenation kinetics during demanding physical activity (Dempsey et al., 2006; Orcioli‐Silva et al., 2024). This perspective is consistent with prior work suggesting that increased respiratory muscle work can evoke sympathetic activation and blood flow redistribution away from locomotor muscles. Moreover, exercise may affect brain regional activation and cerebral oxygenation. These effects occur through adjustments in blood flow, arterial partial pressure of carbon dioxide and blood pressure. By linking the aforementioned mechanisms to observed sex‐specific oxygenation patterns, the study provides an integrative view that connects peripheral and central regulatory responses to exercise. This line of research is particularly promising, as it bridges the gap between exercise and cerebrovascular physiology, offering valuable insight into how sex‐related differences may influence fatigue resistance and endurance capacity.

Based on these sex‐specific oxygenation patterns, the authors point practitioners towards evidence‐based, targeted training interventions. Beyond the findings from the discussed study, available research indicates that the respiratory system may limit exercise performance in females more than in males due to anatomical and physiological differences. Therefore, interventions that enhance respiratory muscle function and attenuate respiratory metaboreflex, such as respiratory muscle training (RMT), are particularly recommended for females (Kowalski et al., 2024). Moreover, the characteristics of fatigue development differ between sexes. Males experience greater peripheral fatigue, likely due to higher sustained workloads and associated increased ischaemia, greater anaerobic contribution and elevated lactate accumulation (Albert et al., 2006; Glace et al., 2013). Consequently, they may benefit more from training methods that promote peripheral adaptations, including high‐intensity interval training (HIIT) to increase mitochondrial efficiency and blood‐flow restriction (BFR) to stimulate vascular remodelling (Atakan et al., 2021; Wortman et al., 2021). Collectively, these strategies highlight the potential for sex‐specific training programmes based on the distinct physiological characteristics identified in the discussed study.

Despite its promise, such integrative research faces several methodological challenges. In particular, sex‐ and ethnicity‐related differences in adipose tissue thickness and structure can affect the penetration and accuracy of NIRS signals, potentially confounding the measurements (Craig et al., 2017). Additionally, NIRS signals are susceptible to interference from skin blood flow during exercise at high intensities (Sudo et al., 2017). Furthermore, measurement is often limited to the prefrontal cortex, which represents a methodological constraint in exercise studies, as this region does not directly reflect activation in motor areas. Moreover, the typical disparities in power output and speed between sexes are inherently linked to differences in mechanical work performed (Billaut & Bishop, 2012). As a result, comparisons made at both submaximal and maximal efforts may not represent equivalent mechanical and physiological loads (Kowalski et al., 2025). These factors underscore the need for careful methodological consideration when designing research and interpreting sex‐based differences in oxygenation and fatigue mechanisms.

Future research could use multimodal approaches that combine NIRS with established measures of cerebral blood flow and cardiopulmonary function to better clarify the mechanisms behind sex‐specific oxygenation patterns. Adding hormonal profiling and controlling for the menstrual cycle phase would further strengthen interpretations of female physiological responses. Moreover, extending such studies to different exercise modalities, intensities, tasks, environments and training statuses seems warranted. Finally, integrating metabolic, neural, cognitive and mechanical indices would enable a more comprehensive understanding of how sex influences the development of fatigue and performance.

## AUTHOR CONTRIBUTIONS

All authors have drafted, revised, and approved the final version of this manuscript and agree to be accountable for all aspects of the work in ensuring that questions related to the accuracy or integrity of any part of the work are appropriately investigated and resolved. All persons designated as authors qualify for authorship, and all those who qualify for authorship are listed.

## CONFLICT OF INTEREST

The authors declare no conflicts of interest.

## FUNDING INFORMATION

No funding was received for this work.
